# Supplementary material for: Association between Antibiotic Consumption and Resistance in Mink Production
Source: Antibiotics (Basel). 2022 Jul 9;11(7):927. doi: 10.3390/antibiotics11070927 (PMC9311663; doi:10.3390/antibiotics11070927)
Supplement: Supplementary file 1 [file antibiotics-11-00927-s001.zip › antibiotics-1796710-supplementary.pdf]

antibiotics

MDPI

Supplementary Materials

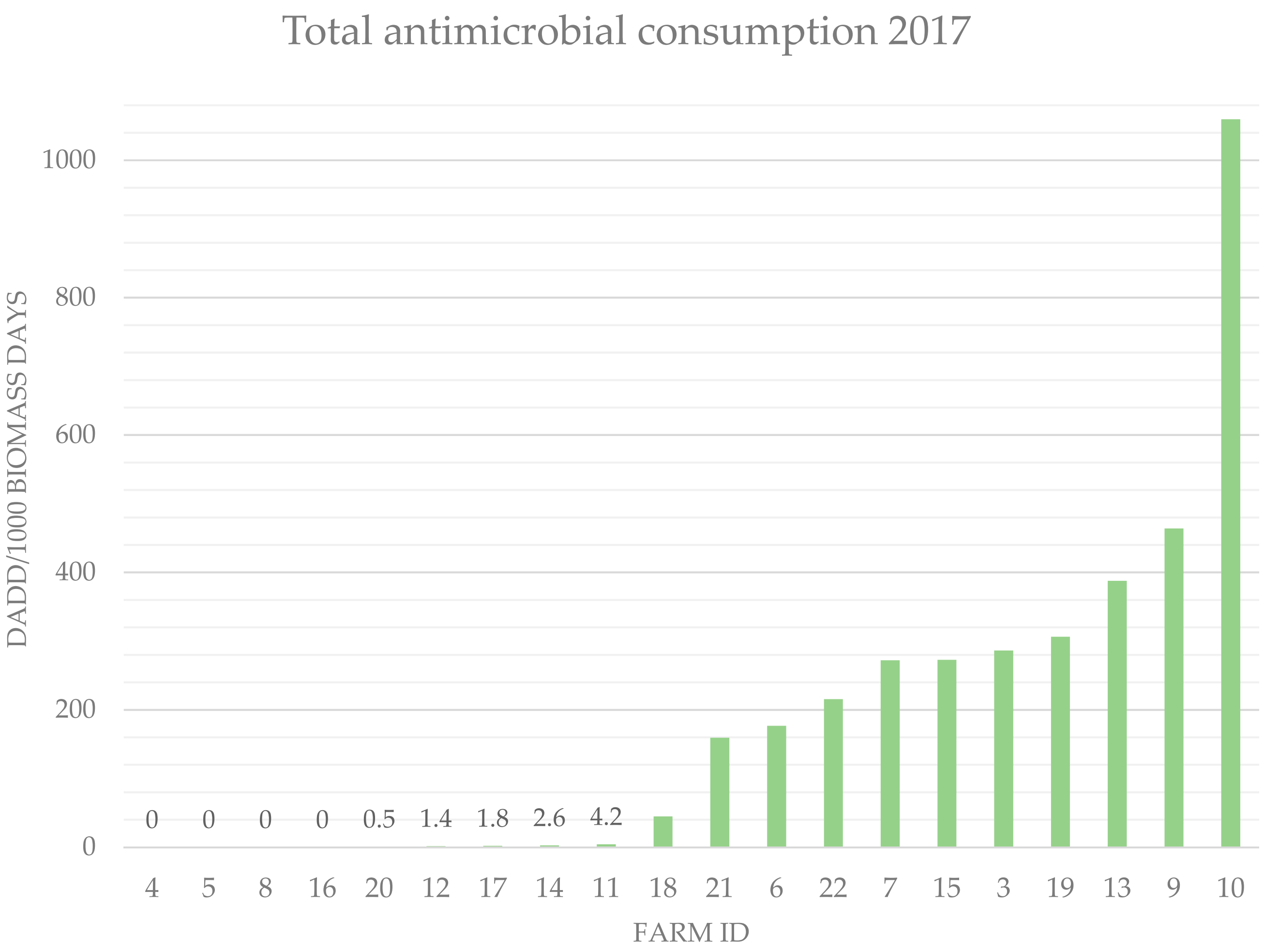

Figure S1. Total antibiotic consumption in 2017. DADD: defined daily animal dose, the assumed average dose needed to treat one kg animal, biomass days calculated as in Jensen et al. [16]

Table S1. Epidemiological cut-off values (ECOFF) (g/L) for *Staphylococcus delphini* and *Escherichia coli.*

Staphylococcus delphini		***Escherichia coli***	
**Antibiotic**	**ECOFF**	**Antibiotic**	**ECOFF**
Cefoxitin	4[a]	Amoxicillin with clavulanic acid (2:1)	8[c]
Chloramphenicol	16 [a]	Ampicillin	8
Ciprofloxacin	1 [a]	Apramycin	16[d]
Erythromycin	1 [a]	Cefotaxime	0.25
Florfenicol	8 [a]	Ceftiofur	1
Gentamicin	2 [a]	Chloramphenicol	16
Benzylpenicillin	O.125 [a]	Ciprofloxacin	0.06
Spectinomycin	128 [b]	Colistin	2
Streptomycin	16 [a]	Florfenicol	16
Sulphamethoxazole	128 [b]	Gentamicin	2
Tetracycline	1 [a]	Nalidixic acid	8
Tiamulin	2 [a]	Neomycin	8
Trimethoprim with Sulphamethoxazole	0.25 [b]	Spectinomycin	64
Trimethoprim	2 [a]	Streptomycin	16
		Sulfamethoxazole	64
		Tetracycline	8
		Trimethoprim	2

[a]ECOFFs adapted from Staphylococcus aureus (www.eucast.org [63]); [b]TECOFF for S. delphini (Nikolaisen et al. 2020 [27]); [c]ECOFF for amoxicillin alone is applied, according to EUCAST MIC distributions and the setting of epidemiological cut-off (ECOFF) values. EUCAST SOP 10.2, 2021 [69]. [d]Tentative ECOFF obtained using data from: 'DANMAP 2016 - Use of antibiotic agents and occurrence of antibiotic resistance in bacteria from food animals, food and humans in Denmark' [67] and Tian et al. 2019 [68].

Table S2. *Staphylococcus delphini* and *Escherichia* coli isolates from Icelandic and Dutch mink farms collected during three sampling rounds in 2018-2019

Staphylococcus delphini								
**Country**	**Sampling round**	**Number of feed producers**	**Number of farms**	**Total number of isolates**	**Number of isolates per farm**	**Proportion of non-wildtype isolates at farm level**		
						Benzylpenicillin	Erythromycin	Tetracycline
Iceland	*Weaning*	-	2	20	10; 10	0.0; 0.0	0.0; 0.0	0.3; 0.4
	Growth	-	2	18	8; 10	0.0; 0.0	0.0; 0.0	0.2; 0.4
	Breeding	-	2	17	7; 10	0.0; 0.0	0.0; 0.0	0.0; 0.4
The Netherlands	*Weaning*	-	2	20	10; 10	0.1; 0.3	0.0; 0.1	1, 1
	Growth	-	2	20	10; 10	0.1; 0.1	0.0; 0.2	1, 1
	Breeding	-	2	20	10; 10	0.0; 0.5	0.0; 0.1	0.9; 1

Escherichia coli								
**Country**	**Sampling round**	**Number of feed producers**	**Number of farms**	**Total number of isolates**	**Number of isolates per farm**	**Proportion of non-wildtype isolates at farm level**		
						Ampicillin	Amoxiclav	Tetracycline
Iceland	*Weaning*	-	2	20	10;10	0.0; 0.2	0.0; 0.0	0.0; 0.1
	Growth	-	2	18	8;10	0.1; 0.4	0.0; 0.0	0.0; 0.4
The	*Weaning*	-	2	16	6;10	0.2; 0.6	0.0; 0.3	0.3; 0.4
Netherlands	*Growth*	-	2	20	10;10	0.6; 0.7	0.2; 0.2	0.5; 0.7

Weaning: bacterial sampling in June 2018. *Growth*: bacterial sampling in October 2018. *Breeding*: bacterial sampling in March 2019. Amoxiclav: Amoxicillin with clavulanic acid (2:1)

Table S3. Non-wildtype (NWT) profiles of susceptibility tested *S. delphini* from the *weaning*, *growth*, and *breeding* samplings from Denmark (NWT profiles n= 66) (tested isolates n=543), Iceland (NWT profiles n= 5) (tested isolates n= 55), and the Netherlands (NWT profiles n=11) (tested isolates n=60). FOX: cefoxitin, CHL: chloramphenicol, CIP: ciprofloxacin, ERY: erythromycin, FFN: florfenicol, GEN: gentamicin, PEN: penicillin, SPE: spectinomycin, STR: streptomycin, SMX: sulfamethoxazole, TET: tetracycline, TIA: tiamulin, TMP: trimethoprim, TMP+SMX: sulfamethoxazole in combination with trimethoprim (19:1).

Denmark

No. of isolates	NWT profile
99	TET
35	PEN-TET
35	SMX-TET
34	ERY- STR
22	TET-ERY-STR
19	ERY
18	PEN
15	PEN-SMX-TET
15	SMX
12	ERY-SPE

12	TMP-SMX-TET
11	SMX-ERY-STR
11	TMP-SMX
8	PEN-TET-ERY-STR
8	PEN-TMP-TET
7	TET-ERY
7	PEN-ERY-STR
7	TMP-TET
5	SMX-TET-ERY-STR
5	TMP
5	TMP-ERY
4	PEN-TMP-SMX-TET
4	SMX-TET-ERY
4	TMP-ERY-STR
3	TET-ERY-SPE
3	PEN-TET-ERY
3	PEN-SMX-ERY-STR
3	PEN-TMP-SMX
2	STR
2	STX-TET
2	PEN-TET-ERY-TIA
2	PEN-TMP-STX-TET
2	TMP-TET-ERY-SPE
2	TMP-TET-ERY-STR
2	TMP-SMX-ERY-SPE
1	ERY-STR-CHL-FFN
1	ERY-STR-FOX
1	PEN-ERY
1	PEN-ERY-SPE
1	PEN-TET-ERY-SPE
1	PEN-TET-FFN
1	PEN-TET-SPE
1	PEN-TET-STR
1	PEN-SMX
1	PEN-SMX-TET-ERY
1	PEN-SMX-TET-ERY-STR
1	PEN-SMX-TET-ERY-STR-GEN-FOX-TIA
1	PEN-TMP
1	PEN-TMP-FFN
1	PEN-TMP-SMX-ERY-STR-FOX-TIA-CIP
1	PEN-TMP-SMX-STR
1	PEN-TMP-SMX-STX-TET
1	PEN-TMP-SMX-TET-ERY
1	PEN-TMP-SMX-TET-ERY-STR

1	SMX-ERY
1	SMX-ERY-SPE
1	SMX-TET-ERY-GEN-FOX-TIA
1	SMX-TET-ERY-SPE
1	TMP-STX-TET
1	TMP-TET-ERY-STR-CHL
1	TMP-TET-SPE-TIA
1	TMP-SMX-ERY-STR
1	TMP-SMX-STX-TET

Iceland

No. of isolates	NWT profile
11	TET
3	TMP
3	SMX-TET
2	SMX
1	TMP-SMX-TET

The Netherlands

No. of isolates	NWT profile
20	TMP-SMX-STX-TET
15	TET
8	PEN-TMP-SMX-STX-TET
5	TMP-STX-TET
4	TMP-TET
2	PEN-TMP-SMX-STX-TET-ERY-STR-TIA
2	TMP-STX-TET-ERY
1	STX-TET-TIA
1	PEN-TMP-STX-TET
1	TMP-SMX
1	TMP-SMX-TET

Table S4: Non-wildtype (NWT) profiles of susceptibility tested *E.coli* from the *weaning* and *growth* samplings from Denmark (NWT profiles n= 89) (tested isolates n=375), Iceland (NWT profiles n= 7) (tested isolates n= 38), and the Netherlands (NWT profiles n=24) (tested isolates n=36). AUG: amoxicillin in combination with clavulanic acid (2:1), AMP: ampicillin, APR: apramycin, FOT: cefotaxime, XNL: ceftiofur, CHL: chloramphenicol, CIP: ciprofloxacin, COL: colistin, FFN: florfenicol, GEN: gentamicin, NAL: nalidixic acid, NEO: neomycin, SPE: spectinomycin, STR: streptomycin, SMX: sulfamethoxazole, TET: tetracycline, TMP: trimethoprim

Denmark

No. of isolates	NWT profile
29	TMP
20	AMP
16	TET
9	SMX-TET
9	AMP-TMP-SMX-TET

9	AMP-STR-SPE
8	AMP-TMP-SMX-STR
8	AMP-SMX-STR-SPE-CHL
7	TMP-CIP
7	AMP-TMP-SMX-TET-STR
5	TMP-TET-STR
5	AMP-TMP-SMX
4	STR
4	AMP-TMP-SMX-STR-SPE
4	AMP-TET-STR
4	AMP-SMX-STR-SPE
4	AMP-AUG-TMP
3	TMP-TET-CIP
3	TMP-TET
3	CIP-NAL
3	CIP
3	AMP-TMP-SMX-TET-SPE
3	AMP-TMP-SMX-TET-CHL-FFN
3	AMP-CIP
2	TMP-CIP-NAL
2	STR-CIP-NAL-XLN
2	AMP-TMP-STR-SPE
2	AMP-TMP-SMX-TET-STR-SPE-CIP-NAL
2	AMP-SMX-TET-STR-SPE
2	AMP-AUG-TMP-SMX-TET-STR-SPE-CHL
2	AMP-AUG-TMP-SMX-TET-STR
2	AMP-AUG-TMP-SMX-STR-SPE
2	AMP-AUG-TMP-SMX-STR
1	AMP-AUG
1	AMP-AUG-STR-SPE-CIP-XLN
1	AMP-AUG-TET-STR
1	AMP-AUG-TET-STR-CHL
1	AMP-AUG-TET-STR-SPE-CHL
1	AMP-AUG-TMP-SMX-STR-CIP
1	AMP-AUG-TMP-SMX-STR-SPE-GEN
1	AMP-AUG-TMP-SMX-TET
1	AMP-AUG-TMP-SMX-TET-STR-SPE-CIP
1	AMP-NAL-XLN
1	AMP-SMX-TET-CIP-NAL
1	AMP-SMX-TET-STR
1	AMP-STR-SPE-CHL
1	AMP-TET-CIP
1	AMP-TET-STR-CHL
1	AMP-TET-STR-NAL-XLN
1	AMP-TMP
1	AMP-TMP-SMX-STR-NEO-CIP-NAL-COL

1	AMP-TMP-SMX-STR-SPE-CHL
1	AMP-TMP-SMX-STR-SPE-XLN-CHL
1	AMP-TMP-SMX-STR-XLN
1	AMP-TMP-SMX-TET-NAL
1	AMP-TMP-SMX-TET-STR-SPE
1	AMP-TMP-STR
1	AMP-TMP-STR-CIP-NAL-XLN
1	AMP-TMP-TET-STR
1	AMP-TMP-TET-STR-CIP
1	AMP-TMP-TET-STR-CIP-NAL-XLN
1	CIP-NAL-XLN
1	SMX
1	SMX-CIP-NAL
1	SMX-STR
1	SMX-TET-CIP-NAL-COL
1	SMX-TET-NEO
1	SMX-TET-STR
1	SMX-TET-STR-NEO-GEN-CIP-NAL-XLN-FOT-COL
1	STR-CIP
1	TET-CIP
1	TET-CIP-NAL
1	TET-STR
1	TMP-CIP-NAL-XLN-FOT
1	TMP-SMX
1	TMP-SMX-TET
1	TMP-SMX-TET-CIP
1	TMP-SMX-TET-XLN
1	TMP-STR
1	TMP-STR-SPE-COL
1	TMP-TET-CIP-NAL
1	TMP-TET-NAL-XLN
1	TMP-TET-STR-CIP-FFN
1	TMP-TET-STR-CIP-NAL-COL
1	TMP-TET-STR-CIP-NAL-XLN-CHL-FFN
1	TMP-XLN
1	XLN

Iceland

No. of isolates	NWT profile
4	AMP
2	TET
1	AMP-SMX-STR
1	AMP-TMP-SMX-TET-STR-CIP-NAL-COL
1	SMX-STR
1	TMP-TET
1	XLN

The Netherlands

No. of isolates	NWT profile
2	AMP-TMP
2	AMP-TMP-SMX
2	AMP-TMP-SMX-STR-SPE
2	SMX-TET-STR-SPE
1	AMP-AUG-SMX-STR-NEO-GEN-APR-CIP-NAL-XLN-FOT-COL
1	AMP-AUG-TMP-SMX-STR-SPE
1	AMP-AUG-TMP-SMX-TET-STR-SPE-NEO-GEN-APR-CIP-NAL-XLN-FOT-COL
1	AMP-TMP-SMX-NEO
1	AMP-TMP-SMX-STR
1	AMP-TMP-SMX-STR-NEO
1	AMP-TMP-SMX-STR-SPE-CIP
1	AMP-TMP-SMX-STR-SPE-CIP-NAL
1	AMP-TMP-SMX-STR-SPE-CIP-NAL-XLN
1	AMP-TMP-SMX-STR-SPE-NEO
1	AMP-TMP-SMX-TET-STR-CIP
1	AMP-TMP-SMX-TET-STR-CIP-NAL
1	AMP-TMP-SMX-TET-STR-SPE-CIP-NAL
1	AMP-TMP-SMX-TET-STR-SPE-GEN-APR-CHL
1	AMP-TMP-SMX-XLN-FOT
1	SMX-TET-STR-NEO-GEN-APR-CIP-NAL-XLN-FOT-CHL-FFN-COL
1	TMP
1	TMP-SMX-STR-SPE-CIP-NAL
1	TMP-SMX-TET-STR-SPE-GEN
1	TMP-SMX-TET-STR-SPE-NEO-GEN-APR-CIP-NAL-XLN-FOT-COL
